# Synthesis and Morphological Control of Biocompatible Fluorescent/Magnetic Janus Nanoparticles Based on the Self-Assembly of Fluorescent Polyurethane and Fe_3_O_4_ Nanoparticles

**DOI:** 10.3390/polym11020272

**Published:** 2019-02-05

**Authors:** Botian Li, Wei Shao, Yanzan Wang, Da Xiao, Yi Xiong, Haimu Ye, Qiong Zhou, Qingjun Jin

**Affiliations:** 1 Department of Materials Science and Engineering, China University of Petroleum, Beijing 102249, China; botian.li@cup.edu.cn (B.L.); shao222333@sina.com (W.S.); wangyanzan_cup@163.com (Y.W.); 18811372167@163.com (D.X.); yehaimu@cup.edu.cn (H.Y.); 2 Research Institute of Chemical Defense, Academy of Military Sciences, Beijing 102205, China; 3 College of Safety and Ocean Engineering, China University of Petroleum, Beijing 102249, China; xiongyi@cup.edu.cn; 4 Key Laboratory of Advanced Materials of Ministry of Education of China, Department of Chemical Engineering, Tsinghua University, Beijing 100084, China

**Keywords:** Janus particle, morphology, fluorescent polyurethane, magnetic nanoparticle, cytocompatibility

## Abstract

Functionalized Janus nanoparticles have received increasing interest due to their anisotropic shape and the particular utility in biomedicine areas. In this work, a simple and efficient method was developed to prepare fluorescent/magnetic composite Janus nanoparticles constituted of fluorescent polyurethane and hydrophobic nano Fe_3_O_4_. Two kinds of fluorescent polyurethane prepolymers were synthesized by the copolymerization of fluorescent dye monomers, and the fluorescent/magnetic nanoparticles were fabricated in one-pot via the process of mini-emulsification and self-assembly. The nanostructures of the resulting composite nanoparticles, including core/shell and Janus structure, could be controlled by the phase separation in assembly process according to the result of transmission electron microscopy, whereas the amount of the nonpolar segments of polyurethane played an important role in the particle morphology. The prominent magnetic and fluorescent properties of the Janus nanoparticles were also confirmed by vibrating magnetometer and confocal laser scanning microscope. Furthermore, the Janus nanoparticles featured excellent dispersity, storage stability, and cytocompatibility, which might benefit their potential application in biomedical areas.

## 1. Introduction

In the past decades, with the development of biomedicine technologies, fluorescent/magnetic nanoparticles (FMNPs) have received increasing attention, as they have shown great superiority in fluorescent labeling and magnetic response [1,2], and could be potentially applied in protein separation [3], drug delivery [4], bio-imaging [5], and so on. Usually, FMNPs consist of fluorescent materials and magnetic nanoparticles such as Fe_3_O_4_ nanocrystals [6,7]. Although many kinds of fluorescent quantum dots derived from Cd and Eu have been emphatically studied to constitute the FMNPs [8,9], these inorganic quantum dots are principally involved in disadvantages of the tedious preparation process and the threat of heavy metal ions with potential toxicity, which limit their further applications for in vivo research [10,11]. Therefore, in most cases, organic fluorescent dyes are considered as the alternative owing to their numerous color species and relatively low cost. Generally, the biological toxicity of dyes could be reduced by the covalent bonding with polymer chain, because the migration of the chromophores is greatly inhibited in the so-called covalently colored polymer; meanwhile, the solvent resistance and the light fastness could also be improved [12,13].

Magnetic Janus particles are of particular utility and interest, which has long been motivated by their enormous applications in micromotors [14,15], panel displays [16], biological imaging [17], and magneto separation [18]. So far, many great efforts have been made to prepare fluorescent/magnetic Janus particles with tunable size, shape, and microstructure [19,20,21], including the microfluidic method [22,23], electrospray method [24], and emulsion method [25]. The two former methods, requiring specialized instruments for preparation, can only produce larger spheres in micro-meter size, while the emulsion method is commonly feasible for the nanoparticles. For example, Gao et. al advocated a microemulsion method to prepare the fluorescent/magnetic Janus nanoparticles (FMJNPs) for magnetolytic therapy of cancer cells using pyrene as a fluorescent probe [26]. However, the addition of small molecule dye introduced the possibility of relatively poor stability and high toxicity. To solve this problem, polymeric fluorescent dyes were introduced in FMJNPs, and the approaches based on the seed emulsion polymerization were developed [27,28], but because of the inhibition of magnetite and fluorophores on polymerization, these methods were confined by low monomer conversion. Furthermore, particle structure was difficult to control. As a consequence, it is still a challenge to develop a facile, versatile, and controllable method to prepare biocompatible FMJNPs.

Polyurethane (PU) is well known as a polymer material with good biocompatibility. Previously, we have reported a series of studies concerning the incorporation of fluorophores into polymer chains to prepare covalently colored PU emulsion [29,30]. In this article, a facile method based on mini-emulsion was developed to fabricate FMJNPs composed of covalently colored fluorescent PU and magnetic nanoparticles. The fluorescent PU prepolymer was synthesized by polymerization of fluorescent monomers derived from 1,8-naphthalimide (NA). After the addition of the hydrophobic Fe_3_O_4_ nanoparticles, the composite nanoparticles with core-shell and Janus structure were produced by the self-assembly method following mini emulsification in one-pot, and their nanostructure could be regulated by changing the non-polar segment ratio of the PU polymer to generate FMJNPs. The resulting Janus particles featured uniform particle size with narrow distribution, as well as promising cytocompatibility and colloidal stability. Moreover, this method was envisaged to be effective and versatile for FMJNPs derived from other hydrophobic polymers, hence it might be potentially applied in the fabrication of various FMJNPs with biocompatibility.

## 2. Materials and Methods 

### 2.1. Materials

We purchased 4-bromo-1,8-naphthalic anhydride, 2-amino-1,3-propanediol, sodium methoxide, diethylamine, cupric acetate monohydrate, dibutyltin dilaurate, sodium dodecyl sulfate (SDS), and 1,2-hexadecanediol (HDO) from Shanghai Aladdin Co. Castor oil (CO), 1,4-butanediol (BDO), isophorone diisocyanate (IPDI), and trimethylol propane (TMP) were supplied by Tianjin Chemicals & Reagents. The solvents, such as tetrahydrofuran (THF), dimethyl sulfoxide (DMSO), chloroform, and methanol, were purchased from Beijing Chemicals. All the reagents were analytically pure and used without further treatment.

### 2.2. Characterizations

The synthesized compounds were characterized using ^1^H NMR (JEOL JNM-ECA600, Japan) and FT-IR (Bruker TENSOR II, Germany). The spectroscopy of fluorescent polymer was measured on a UV/vis spectrometer (Pgeneral T6, Beijing, China) and a luminescence spectrometer (RF-5301PC, Shimadzu, Japan). The nanoparticles were characterized using transmission electron microscopy (TEM) 200kV (JEOL JEM 2100, Japan), SEM (FEI Quanta 200F, Netherlands), X-ray diffraction (XRD) (Bruker AXS, D8 Focus, Germany), and dynamic light scattering (DLS) (Anton-Paar, Litesizer 500, Austria). The measurements of magnetic properties were conducted on a BKT-4500 vibrating sample magnetometer (Xinke, Beijing, China). The conversion of fluoresent dye monomer was determined according to the integral proportion of time dependent curves, which was measured by gel permeation chromatography (GPC) equipped with double detectors (refractive index, UV/vis) (Shimadzu, Japan).

Migration fastness was characterized by extracting the PU latex film with dichloromethane in a Soxhlet extractor for 5 h. The extraction rate was calculated as follows: E = (*d2*/*d1*) × 100%, where *d1* and *d2* denote the dye amount used in the recipe and the extracted dye amount measured by UV analysis, respectively.

Cytocompatibility experiments were carried out using the WST-1 assay. The human intestinal epithelial cells were incubated with the samples for a certain amount of time, after which 10 µL of cell proliferation reagent WST-1 (4-[3-(4-iodophenyl)-2-(4-nitrophenyl)-2H-5-tetrazolio]-1,3-benzene disulfonate) was added to each well and incubated at 37 °C under 7% CO_2_ in an incubator for 2 h. The absorbance was measured in a microplate reader (Bio-Rad) at 450 nm. The optical microscopic pictures were obtained on a microscope (Olympus IX73).

### 2.3. Synthesis of 4-bromo-N-(2-hydroxy-1-hydroxymethylethyl)-1,8-naphthalimide (BHHNA)

A total of 4.17 g (0.015 mol) of 4-bromo-1,8-naphthalic anhydride and 2.43 g (0.027 mol) of 2-amino-13-propanediol were dissolved in 120 mL ethanol, after which the solution was stirred and heated to 78 °C. Then, after 4 h reaction, the solvent was evaporated under reduced pressure at 50 °C. Finally, the yellow crude product was washed three times with deionized water, and then dried in a vacuum oven for 12 h to obtain 4.647 g bright yellow product. Yield: 88%.

^1^H NMR (600 MHz, DMSO-d_6_, TMS, δ): 8.57(d, 1H, NA 5-H), 8.53(d, 1H, NA 7-H), 8.33(d, 1H, NA 2-H), 8.22(d, 1H, NA 3-H), 8.01(d × d, 1H, NA 6-H), 5.22(m, 1H, –C*H*(CH_2_–OH)_2_), 3.82–3.96(m, 4H, –CH(C*H*_2_–OH)_2_). FT-IR (KBr, v/cm^−1^): 3431(O–H stretch), 1726(C=O stretch), 1592, 1500 (NA skeleton vibration) (Figure S1).

### 2.4. Synthesis of 4-methoxyl-N-(2-hydroxy-1-hydroxymethylethyl)-1,8-naphthalimide (MHHNA)

3.5 g (0.01 mol) BHHNA and 1.62 g (0.03 mol) sodium methoxide were added to a flask containing 60 mL methanol, the mixture was stirred and heated to 65 °C, after 4 h reaction the solvent was evaporated under reduced pressure at 40 °C, resulting the white solid. Afterward, the crude product was washed for three times with deionized water, then dried in a vacuum oven for 12 h to give 2.35 g product. Yield: 78%.

^1^H NMR (600 MHz, DMSO-d_6_, TMS, δ): 8.53 (d, 1H, NA 5-H), 8.49 (d, 1H, NA 7-H), 8.45 (d, 1H, NA 2-H), 7.83 (d × d, 1H, NA 6-H), 7.33 (d, 1H, NA 3-H), 5.22 (m, 1H, –C*H*(CH_2_–OH)_2_), 4.13 (s, 3H, –O–CH_3_), 3.81–3.94 (m, 4H, –CH(C*H*_2_–OH)_2_) (Figure S2). FT-IR (KBr, v/cm^−1^): 3431 (O–H stretch), 2937 (CH_3_, C–H stretch), 1688 (C=O stretch) (Figure S3).

### 2.5. Synthesis of 4-diethylamino-N-(2-hydroxy-1-hydroxymethylethyl)-1,8-naphthalimide (DHHNA)

A total of 2.57 g (0.0075 mol) of BHHNA, 5.47 g (0.075 mol) of diethylamine, and 0.4 g (0.002 mol) of cupric acetate monohydrate were dissolved in 20 mL DMSO, and the solution was stirred and heated to 120 °C. Then, after 12 h reaction, it was cooled to room temperature and precipitated in 150 mL deionized water. The solid was filtered and washed three times, and then dried in vacuum. The crude product was purified by silica column chromatography using a mixture of dichloromethane/ethyl acetate (1:3) as eluent to give 1.165 g yellow product. Yield: 45%.

^1^H NMR (600 MHz, DMSO-d_6_, TMS, δ): 8.45 (d, 1H, NA 5-H), 8.43 (d, 1H, NA 7-H), 8.36 (d, 1H, NA 2-H), 7.78 (d × d, 1H, NA 6-H), 7.35 (d, 1H, NA 3-H), 5.22 (m, 1H, –C*H*(CH_2_–OH)_2_), 3.78–3.96 (m, 4H, –CH(C*H*_2_–OH)_2_), 3.39 (q, 4H, –N–(C*H*_2_–CH_3_)_2_), 1.10 (t, 6H, –N–(CH_2_–C*H*_3_)_2_) (Figure S4). FT-IR (KBr, v/cm–^1^): 3451 (O–H stretch), 2965, 2884 (saturated C–H stretch), 1689 (C=O stretch), 1589, 1480 (NA skeleton vibration) (Figure S5).

### 2.6. Synthesis of PU Prepolymer

According to the recipe listed in Table 1, IPDI, hydroxyl chain extenders, and THF were charged into a three-necked flask equipped with thermometer, reflux condenser, and electric mechanical stirrer, and one drop of dibutyltin dilaurate was added as catalyst. The flask was maintained at 65 °C in an oil bath for 4 h under stirring, following which the reaction system was cooled to 50 °C, and PU prepolymers were obtained after removal of THF by rotary evaporation under reduced pressure.

### 2.7. Preparation of Composite Nanoparticles

The hydrophobic magnetic nanoparticles (HMNPs) were synthesized by the thermal decomposition of iron oleate in the presence of oleic acid, according to the literature [31].

In the principal fabrication procedure of composite nanoparticles, taking PTCP-20 as an example, 20 mg of HMNPs and 80 mg of PU-TMP were dissolved in 2.8 g CHCl_3_. After the addition of 5 mL SDS aqueous solution (0.6 mg/mL), the mixture was emulsified for 10 min by ultrasonication (200 W) in an ice bath to give a mini-emulsion. Subsequently, the emulsion was slowly evaporated in a rotary evaporator at 55 °C for 15 min until no chloroform vaporized, resulting a dark yellow latex of PTCP-20 (20% HMNPs content). PCCP-*c*, PMCP-*c*, and PDCP-*c* were similarly prepared by utilizing PU-CO, PU-MHHNA, and PU-DHHNA, respectively, wherein *c* denoted HMNPs content (*c* = 0, 10, 20, 30) (Table 2).

## 3. Results and Discussion

### 3.1. Characterization of Fluorescent Polyurethane

**Scheme 1:**
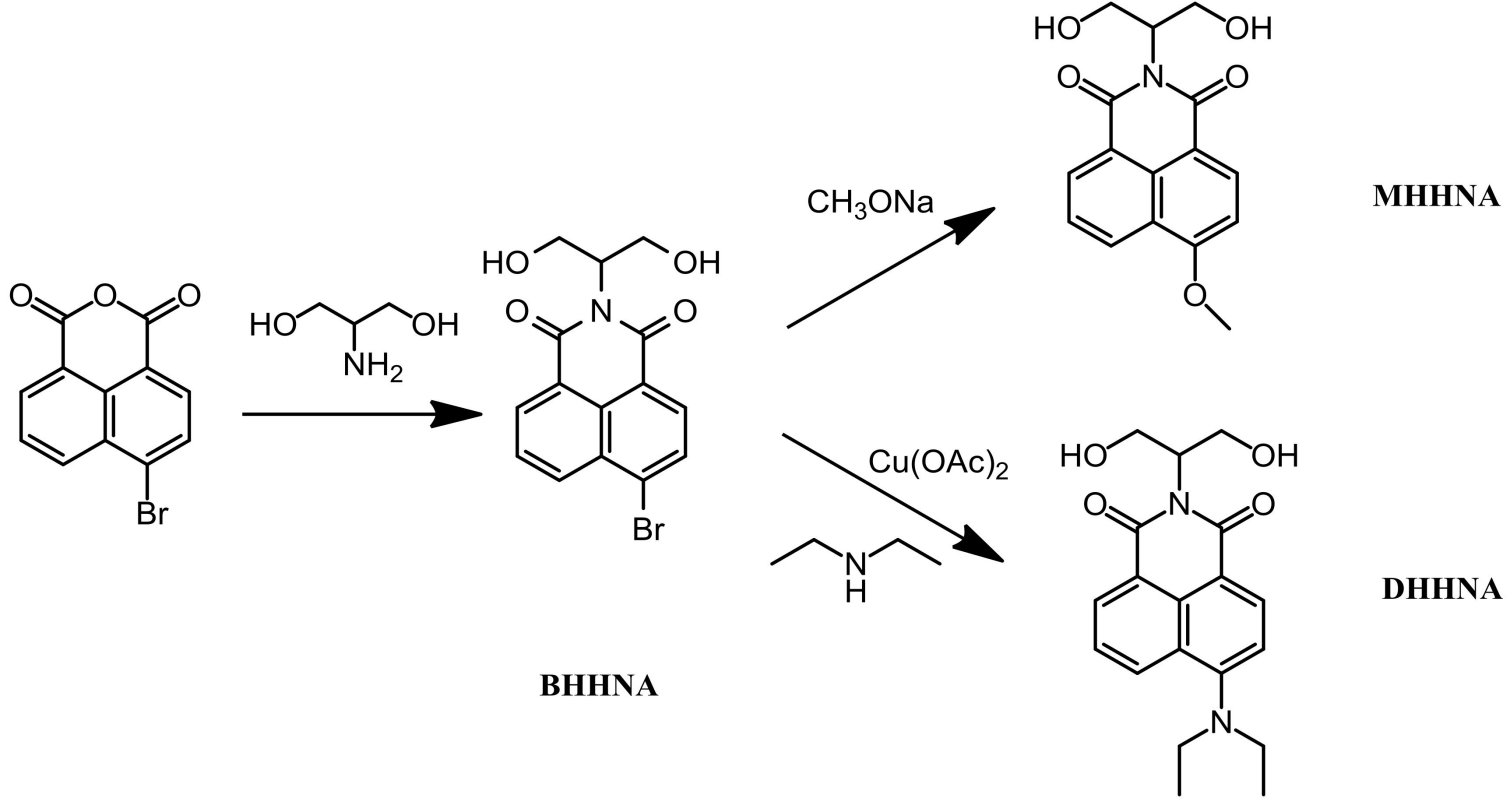
Synthetic route of fluorescent monomers. BHHNA—4-bromo-N-(2-hydroxy-1-hydroxymethylethyl)-1,8-naphthalimide; MHHNA—4-methoxyl-N-(2-hydroxy-1-hydroxymethylethyl)-1,8-naphthalimide; DHHNA—4-diethylamino-N-(2-hydroxy-1-hydroxymethylethyl)-1,8-naphthalimide.

**Scheme 2:**
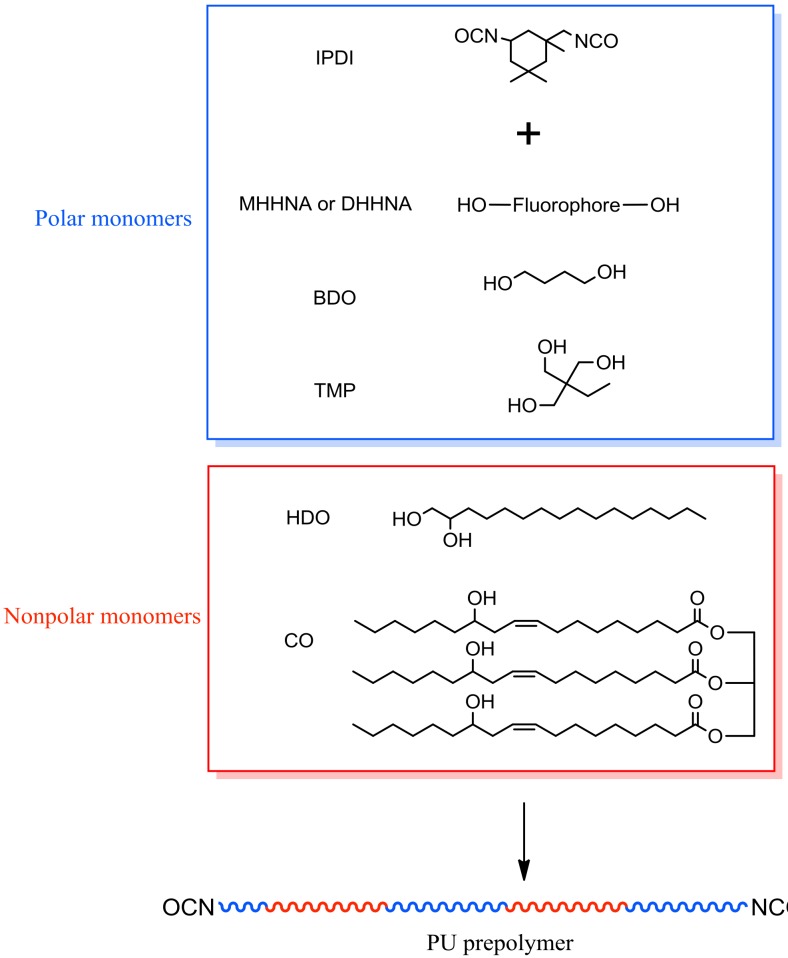
Illustration of polyurethane (PU) prepolymer synthesis. TMP—trimethylol propane; CO—castor oil; IPDI—isophorone diisocyanate; HDO—1,2-hexadecanediol; BDO—1,4-butanediol.

As illustrated in Scheme 1, two fluorescent dyes (MHHNA, DHHNA) were synthesized by a two-step procedure of imidization and nucleophilic substitution. Both of them consisted of 1,8-naphthalimide chromophore, but offered contrasting fluorescent colors due to the different electron-donating substituents. These dyes were employed in the synthesis of polyurethane by reacting with isocyanate group as chain extenders, thus introducing the fluorescent units into macromolecular chain to produce covalently colored fluorescent PU prepolymers containing isocyanate end group (Scheme 2). The polymerization conversion of fluorescent monomers measured by GPC method showed results of 98.5% (MHHNA) and 97.3% (DHHNA), suggesting that most of the fluorescent chromophores were covalently grafted on PU (Figures S6 and S7), and that the covalently colored fluorescent prepolymer was successfully prepared. To characterize the migration fastness of fluorophores, the fluorescent PU films PMCP-0 and PDCP-0 were extracted in dichloromethane for 5 h, both giving extraction rates below 2%. Because dichloromethane was a good solvent for MHHNA and DHHNA, it could be deduced that the covalent bonding with the polymer chain significantly improved the migration fastness of the fluorophore.

**Figure 1:**
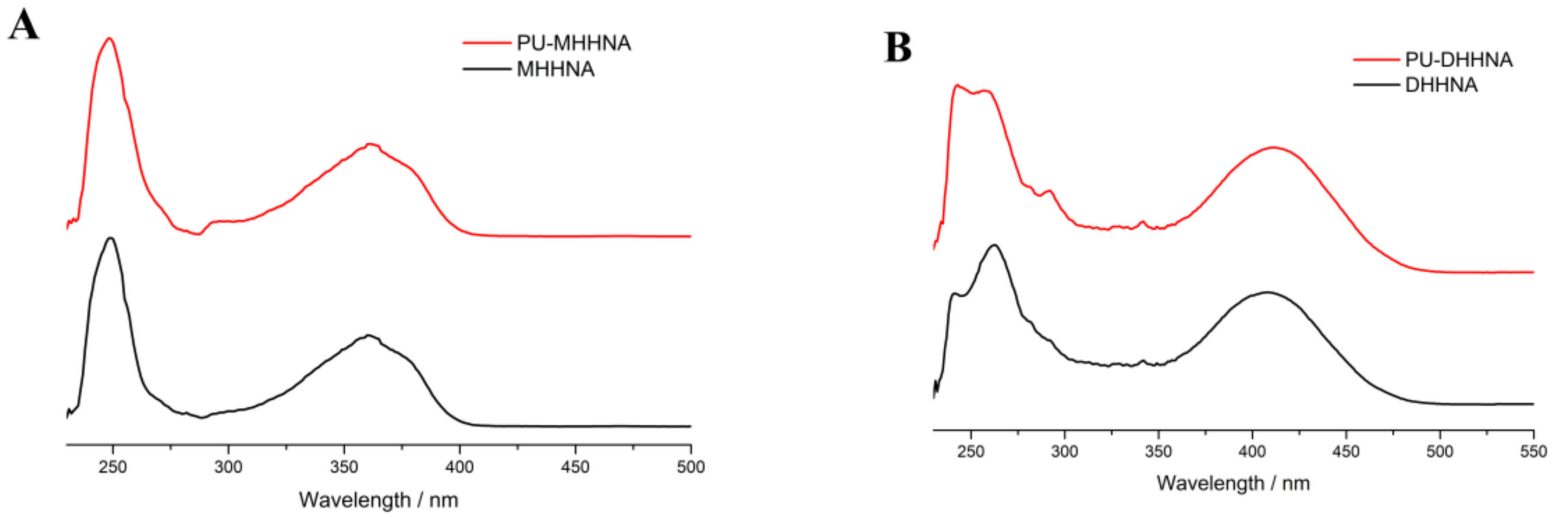
UV/vis spectra of fluorescent dye (4-methoxyl-N-(2-hydroxy-1-hydroxymethylethyl)-1,8-naphthalimide (MHHNA), 4-diethylamino-N-(2-hydroxy-1-hydroxymethylethyl)-1,8-naphthalimide (DHHNA)) and corresponding fluorescent polyurethane (PU) prepolymer in ethyl acetate solution.

**Figure 2:**
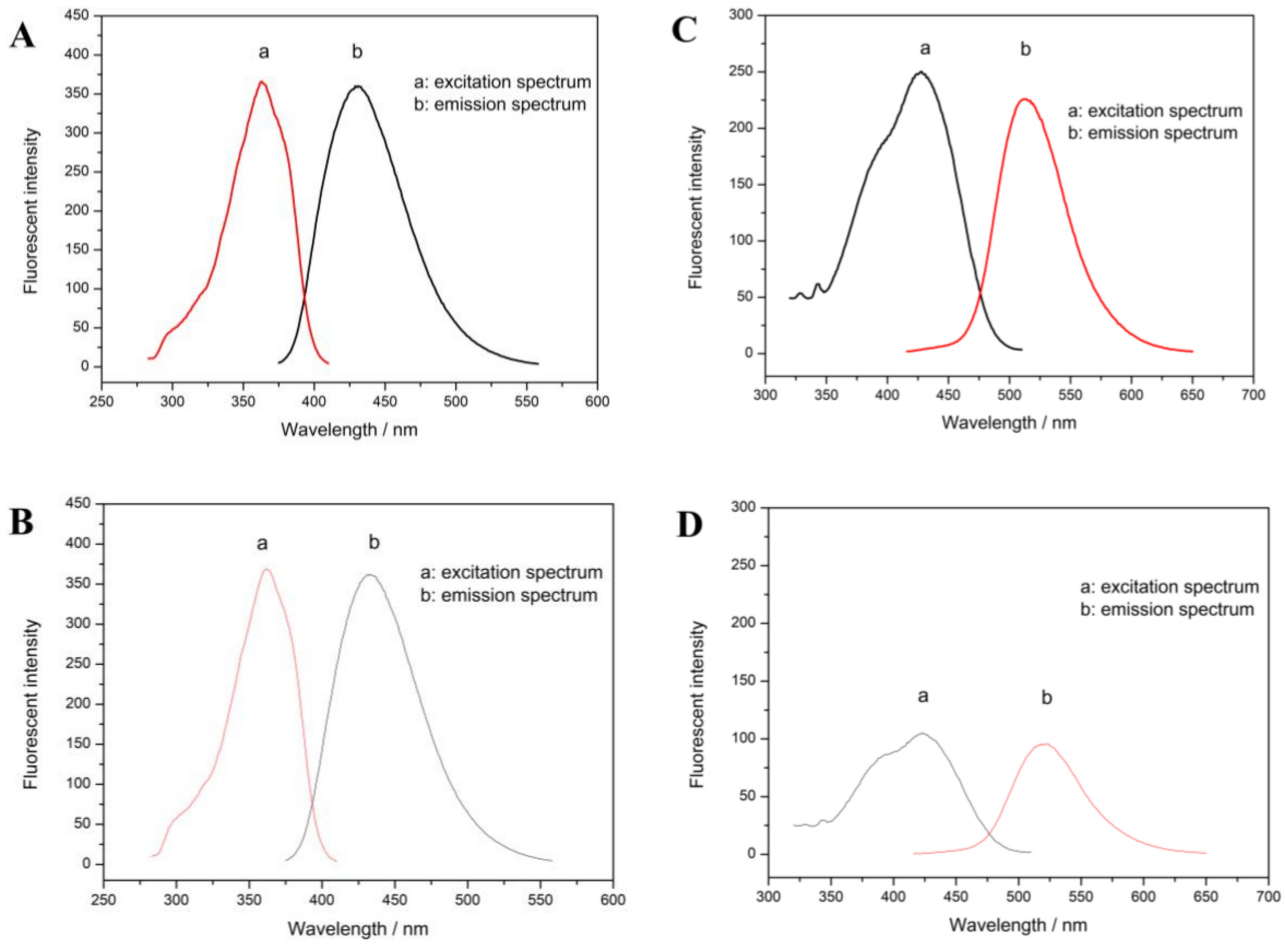
Fluorescence spectra of (**A**) PU-MHHNA, (**B**) MHHNA, (**C**) PU-DHHNA, and (**D**) DHHNA in ethyl acetate solution.

The UV/vis spectroscopy of the fluorescent monomers and the fluorescent prepolymers were determined at 6.6 × 10^−2^ mM fluorophore concentration in the solutions of both monomer and prepolymer. MHHNA and PU-MHHNA were colorless with almost no absorption in the visible light range (>400 nm), while DHHNA and PU-DHHNA were yellow under visible light because of their red-shifted n→π^*^ absorption band. As shown in Figure 1, the UV/vis absorption spectra of PU-MHHNA were almost consistent with that of MHHNA with the maximum absorption wavelength (λ_max_) at 362 nm, and DHHNA and PU-DHHNA had the same UV/vis profile with λ_max_ at 419 nm. These results indicated that the fluorescent chromophores did not change their chemical structures during polymerization. In the study of fluorescent properties, it was revealed that the fluorescent spectra of MHHNA and PU-MHHNA were completely identical at 1.66 × 10^−3^ mM fluorophore concentration, their fluorescent excitation and emission spectra presented a good mirror-image relationship, as well as their maximum fluorescent excitation wavelength (λ_ex_) and emission wavelength (λ_em_) exhibited at 362 and 433 nm, respectively, with Stokes shift of 71 nm (Figure 2A,B). Using quinine sulfate as reference, the fluorescence quantum yields of MHHNA and PU-MHNNA were measured to be 0.71. Meanwhile, λ_ex_ (421 nm) and λ_em_ (522 nm) of PU-DHHNA were also the same as that of DHHNA, thus generating a larger Stokes shift (101 nm), because the diethylamino group possessed stronger electron donating ability than the methoxy group, causing a higher intramolecular charge transfer rate and a smaller energy gap between HOMO and LUMO [32,33]. Furthermore, it is worth noting that at the same fluorophore concentration (3 × 10^–2^ mM), PU-DHHNA displayed enhanced fluorescence intensity in emission and excitation spectra, and its quantum yield (0.15) was considerably increased compared with DHHNA (0.08) (Figure 2C,D). This result might be ascribed to the fact that DHHNA monomers probably underwent aggregation-caused quenching (ACQ) in relatively higher concentration by interacting with each other through intermolecular charge transfer interaction, yet the steric hindrance of PU-DHHNA chain hindered the ACQ effect between the fluorophores, thereby increasing the fluorescence quantum yield. Consequently, the polymerization of fluorescent monomers could not only prevent the migration of fluorophore, but also increase the fluorescence intensity.

### 3.2. Fabrication of Composite Nanoparticles

**Figure 3:**
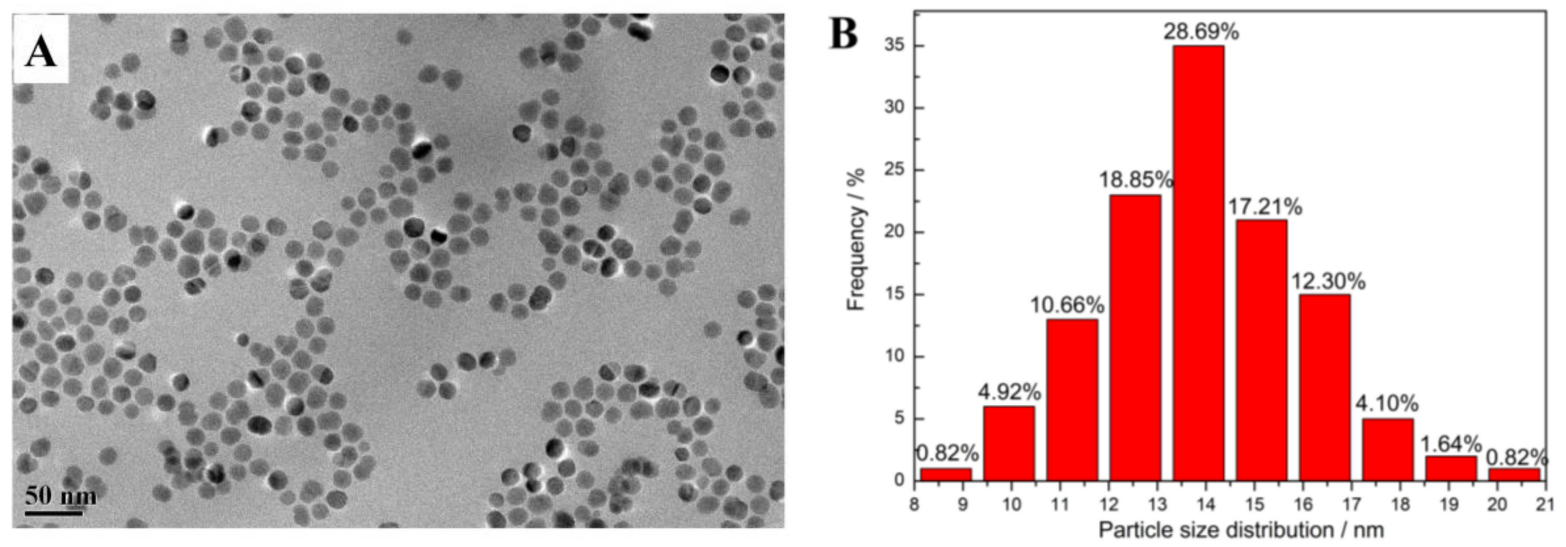
(**A**) Transmission electron microscopy (TEM) image of the hydrophobic magnetic nanoparticles (HMNPs) and (**B**) the frequency of the particle size.

**Figure 4:**
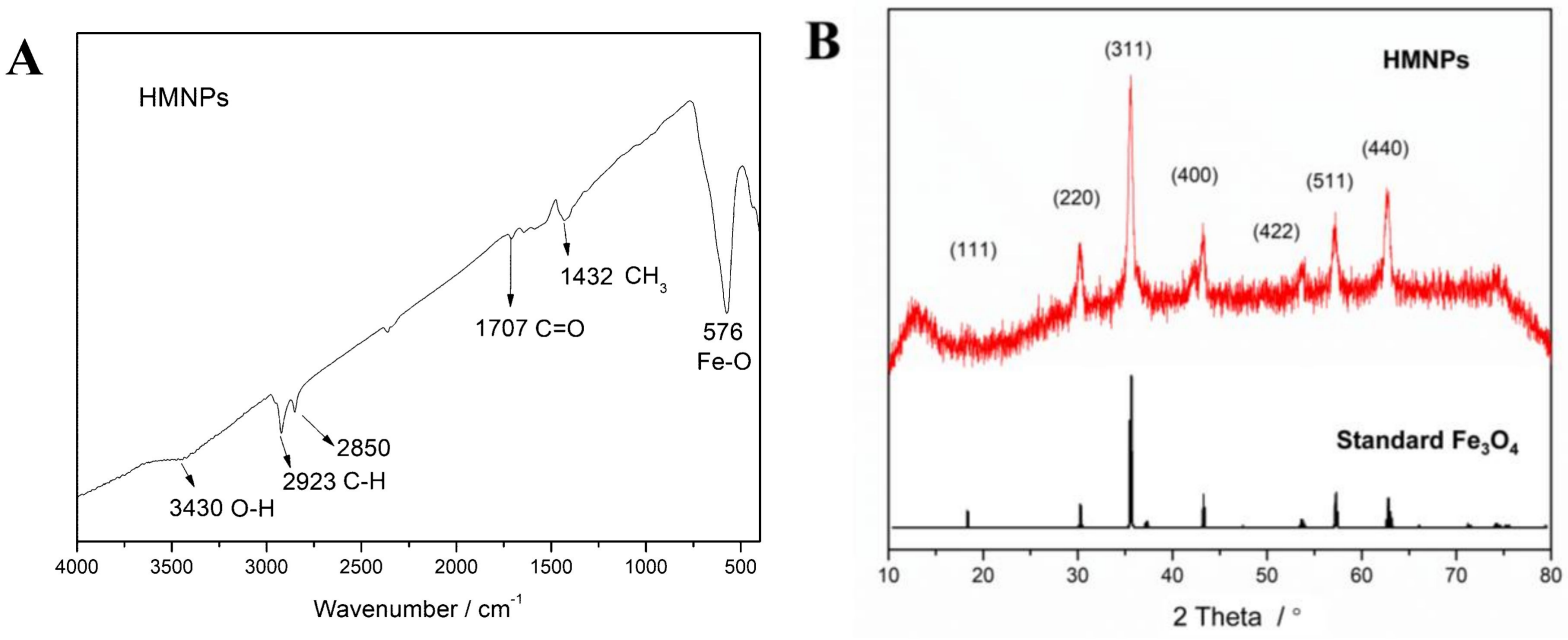
(**A**) FT-IR spectrum of HMNPs and (**B**) X-ray diffraction (XRD) pattern of HMNPs compared with standard XRD of Fe_3_O_4_.

Hydrophobic magnetic nanoparticles prepared by thermal decomposition of ferric oleate had good solubility in nonpolar solvents such as toluene, chloroform, or n-hexane, and could form magnetic liquid in these solvents through surface solvation, while they expectedly precipitated in polar solvents such as ethanol or water because of their poor solubility (Figure S8). It was believed that the surface coating of oleic acid by coordination imparted hydrophobicity to the magnetic nanoparticles. From TEM observation, HMNPs exhibited relatively uniform spherical particles, their particle size was analyzed by nano-measure software to give the frequency of diameter in the TEM image. As indicated in Figure 3, the size of HMNPs was mostly in the range of 11–17 nm with an approximately normal distribution. In the FT-IR spectrum (Figure 4A), the absorption peaks located at 2923 and 2850 cm^−1^ should be assigned to the saturated C–H stretching, the signals at 1707 cm^−1^ stemmed from the oleate C=O vibration, and the intensive peak at 576 cm^−1^ could be attributed to the stretching vibration of the Fe–O bond, suggesting that HMNPs were composed of magnetite and oleic acid. By comparing with the standard XRD pattern, one could ascertain that the crystal structure of HMNPs was the same as that of magnetite (Figure 4B), except that the peaks of HMNPs were significantly broadened because of the grain refinement. According to the Debye–Scherrer formula—D = K·λ/B·cosθ, where Scherrer constant K = 0.89, X-ray wavelength λ = 0.15406 nm, diffraction angle θ = 17.8° (311), and half-peak width B = 0.0091 rad (311)—the average grain size D could be calculated as 15.8 nm, which was slightly larger than the diameter of the most probable distribution (14 nm) in the TEM result, which implied that some particles with a larger size (>22 nm) might not be counted by TEM measurement. Because the critical dimension of paramagnetic Fe_3_O_4_ (single domain) was about 30 nm at room temperature [34], that part of the magnetic particles with a larger size could generate the coercivity in HMNPs; accordingly, the remanence occurred when the external magnetic field was removed (Figure 8).

The composite particles were fabricated via two steps including mini-emulsification and self-assembly in solvent evaporation. First, the PU prepolymer and HMNPs were dissolved in chloroform to form a dilute solution with low viscosity, then an aqueous solution of very few SDS was added and the mixture was homogenized by ultrasonication to obtain mini-emulsion, finally the solvent was evaporated under reduced pressure and the composite particles were formed through the assembly of polyurethane and HMNPs. As chloroform volatilized, PU prepolymers were exposed to the aqueous phase, then their molecular weight gradually increased by the chain extending reaction of isocyanate group and H_2_O. Simultaneously, because HMNPs were hydrophobic and extremely arduous to diffuse into the aqueous phase, they would only remain in the nanospheres with PU macromolecules to form inorganic–organic nanocomposites. Notably, to promote the stability of mini-emulsion, it was usually necessary to employ hydrophobic agents to balance the Laplace pressure and prevent the Ostwald ripening [35]; however, in this work, HMNPs in droplet were intrinsically hydrophobic, thus a narrow distribution of particle size could be achieved without the addition of other hydrophobic agents such as hexadecane or hexadecanol.

### 3.3. Nanostructure of Composite Nanoparticles

**Figure 5:**
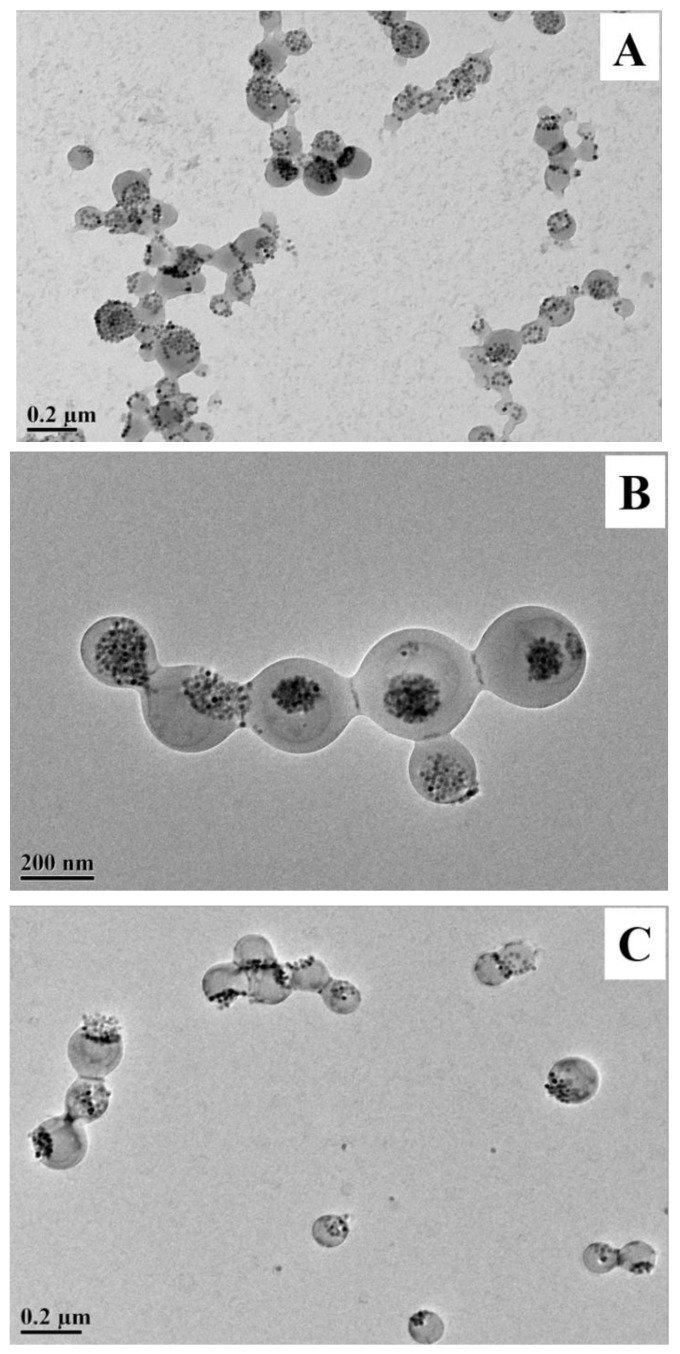

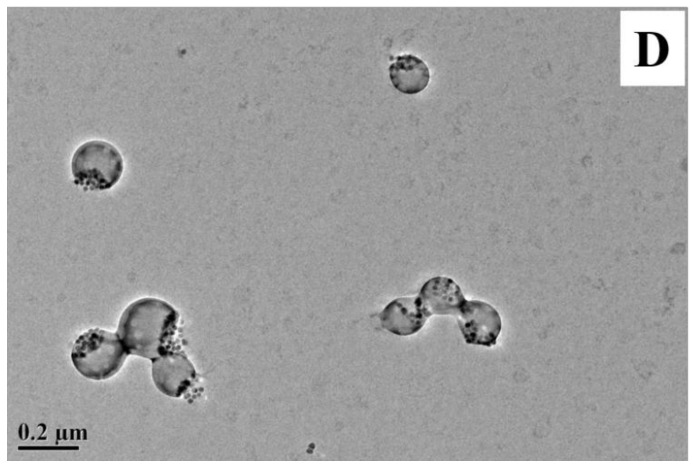
TEM images of (**A**) PU-trimethylol propane (TMP) magnetic composite nanoparticle (PTCP-20), (**B**) PU-castor oil (CO) magnetic composite nanoparticle (PCCP-20), (**C**) PU-MHHNA magnetic composite nanoparticle (PMCP-20), and (**D**) PU-DHHNA magnetic composite nanoparticle (PDCP-20).

**Figure 6:**
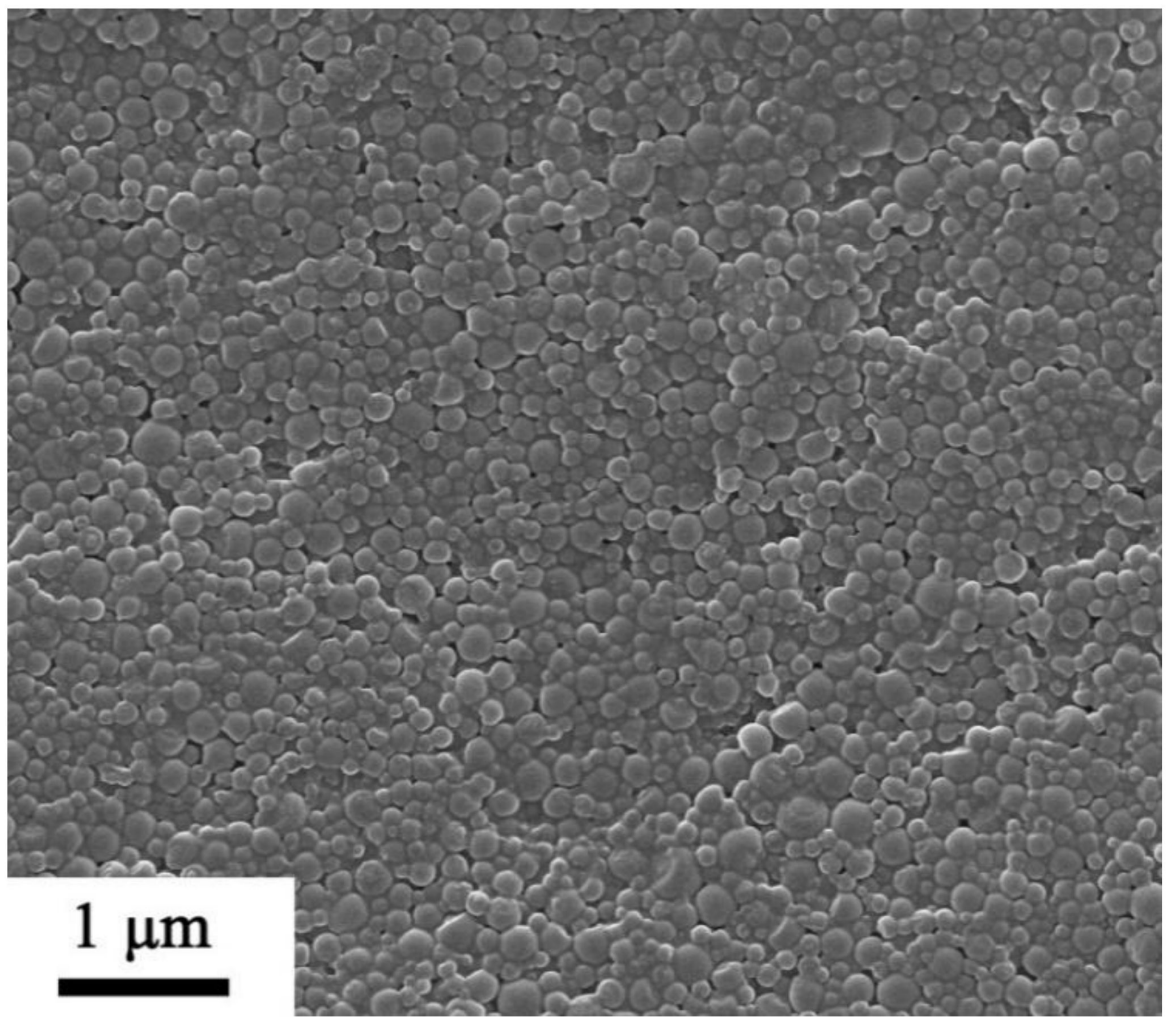
SEM image of PMCP-20.

To investigate the morphology and the nanostructure, the composite nanoparticles with different ingredients were investigated using the TEM and SEM methods, and it was revealed that the proportion of PU nonpolar and polar segments played an important role in the assembly structure of nanocomposites. PU-TMP without nonpolar segments was first tried in the fabrication of PTCP-20. As shown in Figure 5A, the HMNPs could not be effectively encapsulated in the nanospheres and they mostly distributed on the surface, thus the further aggregation of HMNPs resulted in the precipitation and delamination of latex. In contrast, after the addition of PU-CO containing unsaturated hydrophobic segments, most HMNPs were embedded inside the nanoparticles PCCP-20 (Figure 5B), and the storage stability of the latex was obviously improved. However, it was shown in Figure 5B and Table 3 that the particle size of PCCP-20 prepared from PU-CO was much larger because of the increase of hydrophobicity in composite particles, and the size distribution of PCCP-20 became broad, which might be ascribed to the fact that abundant multifunctional monomer CO increased the crosslinking degree of the prepolymer, which not only broadened the molecular weight distribution, but also increased the viscosity of the organic phase in mini-emulsion to retard homogeneous emulsification. To solve this problem, a bifunctional diol HDO was employed to introduce the hydrophobic segment into PU, replacing a proportion of CO in the recipe of PMCP and PDCP (Table 1). In Figure 5C, it was seen that almost all nanoparticles of PMCP-20 displayed manifest Janus-type structure because of the phase separation, and HMNPs remained inside the nanoparticles, indicating the formation of FMJNPs. Similarly, the Janus nanostructures of PDCP-20 were completely consistent with PDMP-2, as shown in Figure 5D. Moreover, the SEM image of PMCP-20 presented in Figure 6 demonstrated the FMJNPs were well distributed and their smooth surface inferred that HMNPs located in the particles rather than on the surface. The investigation of the colloidal properties measured by DLS further revealed that the Janus nanoparticles of PMCP-20 and PDCP-20 enjoyed a uniform particle size distribution with reduced polydispersity index in comparison with PCCP-20 (Table 3).

**Scheme 3:**
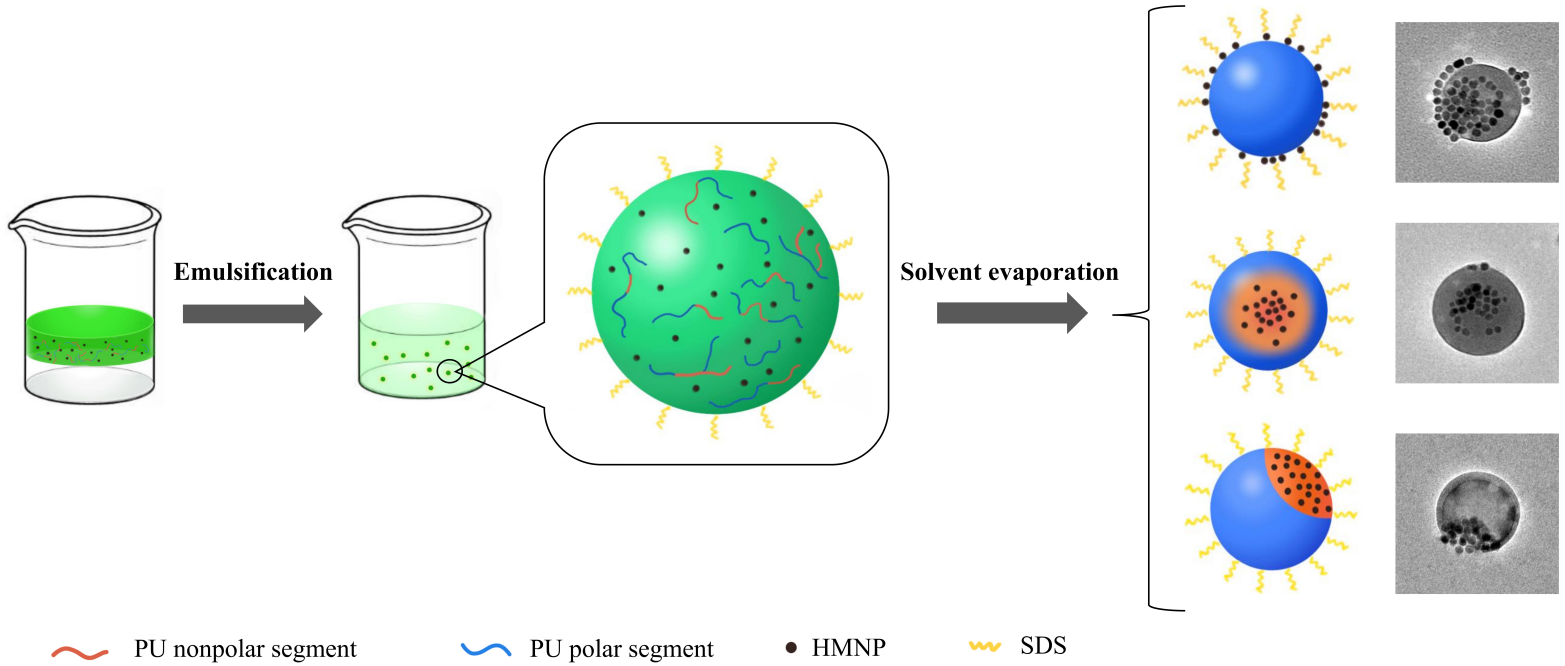
Illustration of the preparation of the composite nanoparticles. HMNP—hydrophobic magnetic nanoparticle; SDS—sodium dodecyl sulfate.

From these results, it was inferred that the compatibility between HMNPs and PU was of importance in the formation of stable composite nanoparticles by reducing the interface energy, the compatibility arose from the hydrophobic interaction between PU nonpolar segments and oleic acid coated HMNPs. On the basis of the different proportions of polar and nonpolar segments in PU, three structures of composite nanoparticles could be formed in the assembly process (Scheme 3), and are classified as follows: (1) Nanoparticles coated with HMNPs, in which PU constituted of all polar segment had almost no affinity with HMNPs, leading to the severe phase separation, which excluded the HMNPs out of the sphere. With the aid of surfactant, the hydrophobic nano Fe_3_O_4_ could only disperse at the solid–water interface, and its combination with the nanoparticle was instable (Figure 5A). (2) Nanoparticles with HMNPs/PU as core/shell structure, in which PU consisted of considerable nonpolar segments from CO or HDO, which suggested it promising interfacial compatibility with HMNPs. Because of their hydrophobicity, the nonpolar segments of PU and HMNPs were inclined to be distributed in the core of composite nanoparticles. (3) Janus nanoparticles, where PU contained fewer nonpolar segments. The polymer phase was less compatible with HMNPs, thereby the phase separation of PU polar polymer and HMNPs occurred during the solvent evaporation process, resulting in the Janus-type particles. Accordingly, it was beneficial to utilize a suitable amount of nonpolar chain extender in the synthesis of PU prepolymer for the preparation of FMJNPs. 

**Figure 7:**
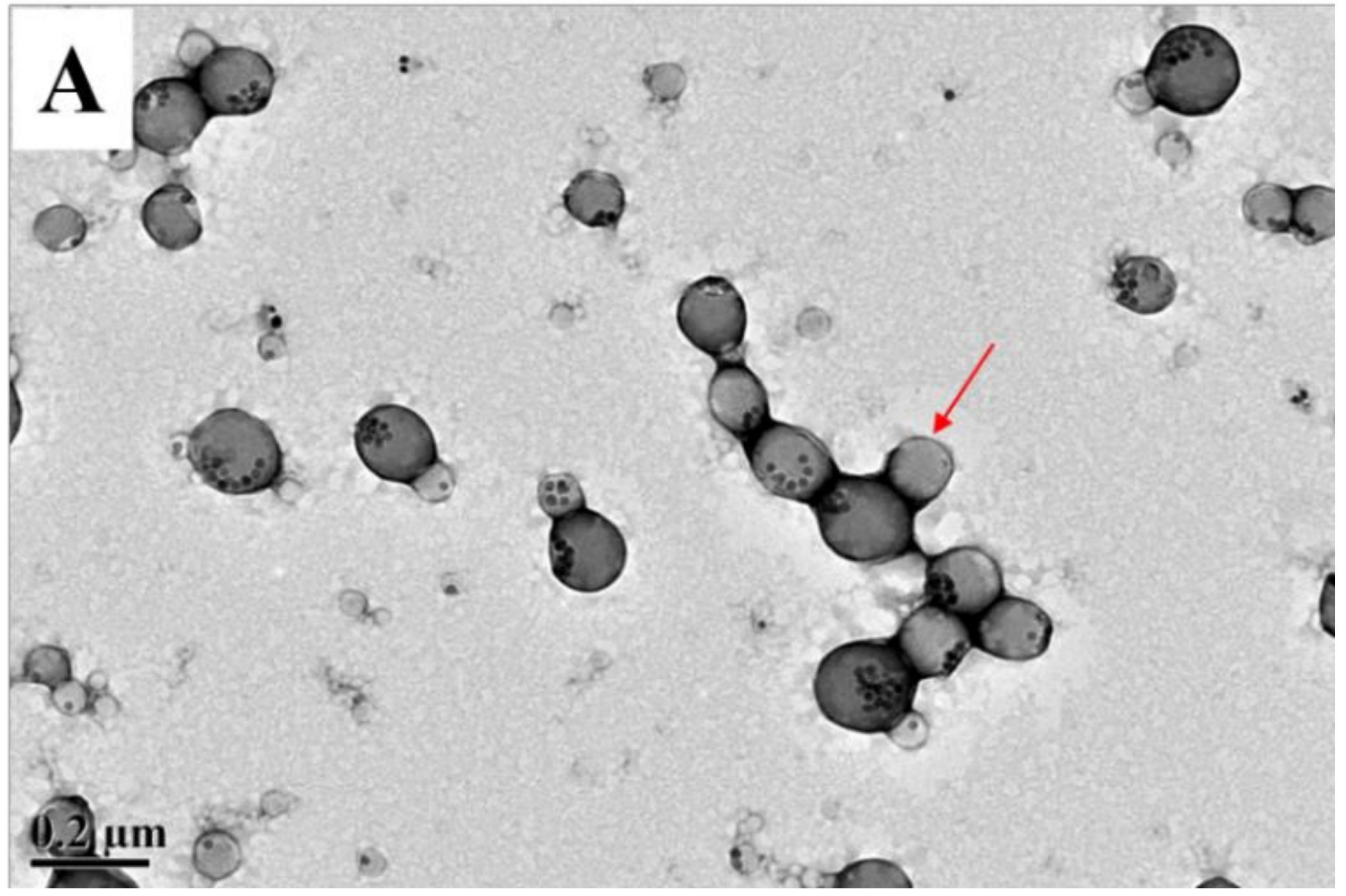

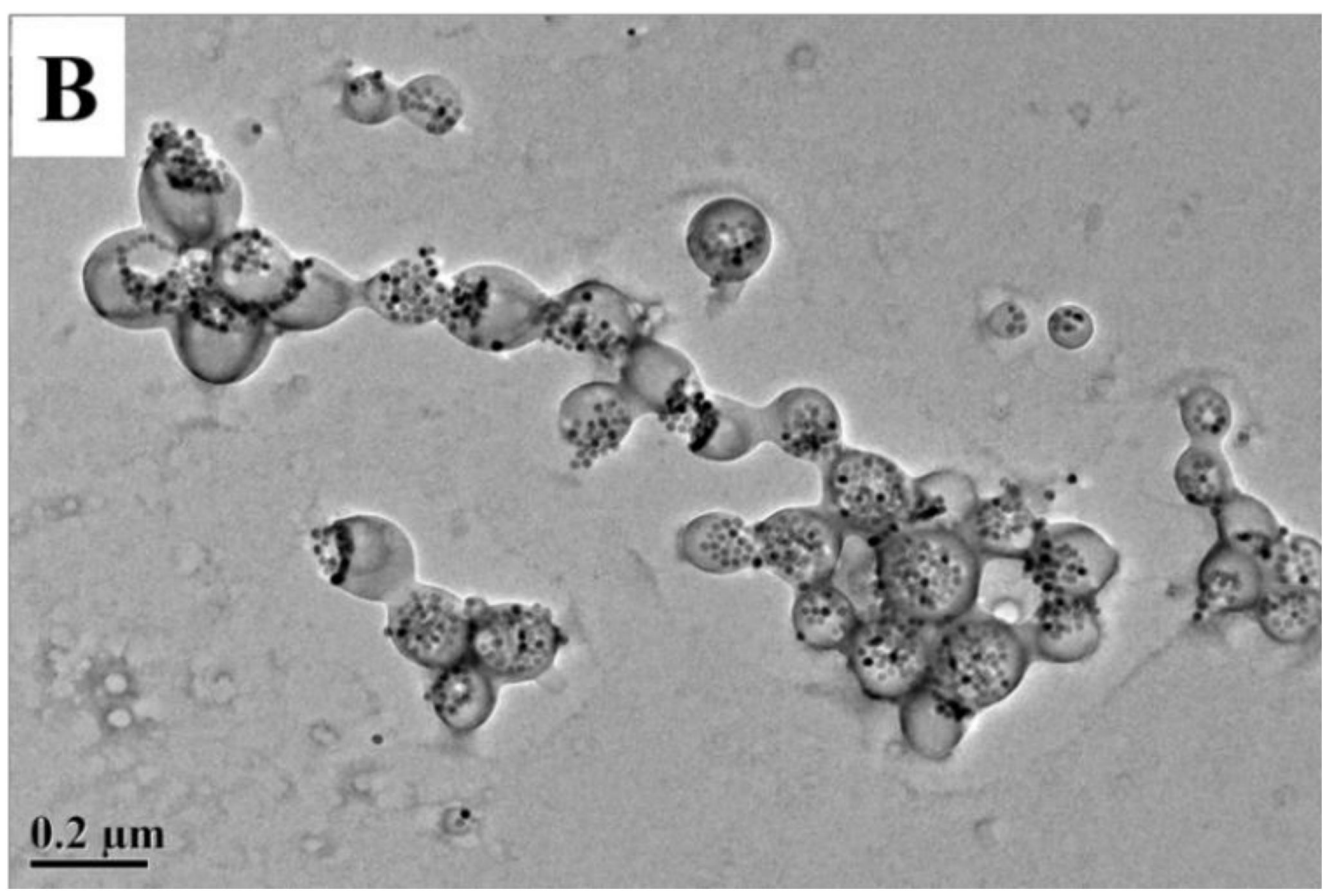
TEM images of (**A**) PMCP-10 and (**B**) PMCP-30.

Furthermore, the particle structures of PMCP with different HMNP contents were examined by TEM. In Figure 7A, HMNPs accounted for only 10% of the total weight in PMCP-10, and most of them were encapsulated in the nanoparticles, despite the fact that they were not evenly dispersed because of the phase separation. The polymer spheres without the composition of HMNPs could also be distinguished as marked by the arrow. With HMNP content increased to 30% (PMCP-30), most of the nanoparticles were entirely filled with HMNPs, triggering a lower ratio of Janus nanoparticles (Figure 7B). The recipe with HMNPs content higher than 30% was still applicable in preparation, but the stability of the product decreased with the increasing HMNPs. Therefore, with the optimum HMNP content of 20% for Janus particles, PMCP-20 and PDCP-20 were selected for further study, and their fluorescent magnetic latexes both displayed excellent storage stability, showing neither the demulsification of the latex nor the precipitation of Fe_3_O_4_ during five months of storage.

### 3.4. Magnetic and Fluorescent Properties of Composite Nanoparticles

**Figure 8:**
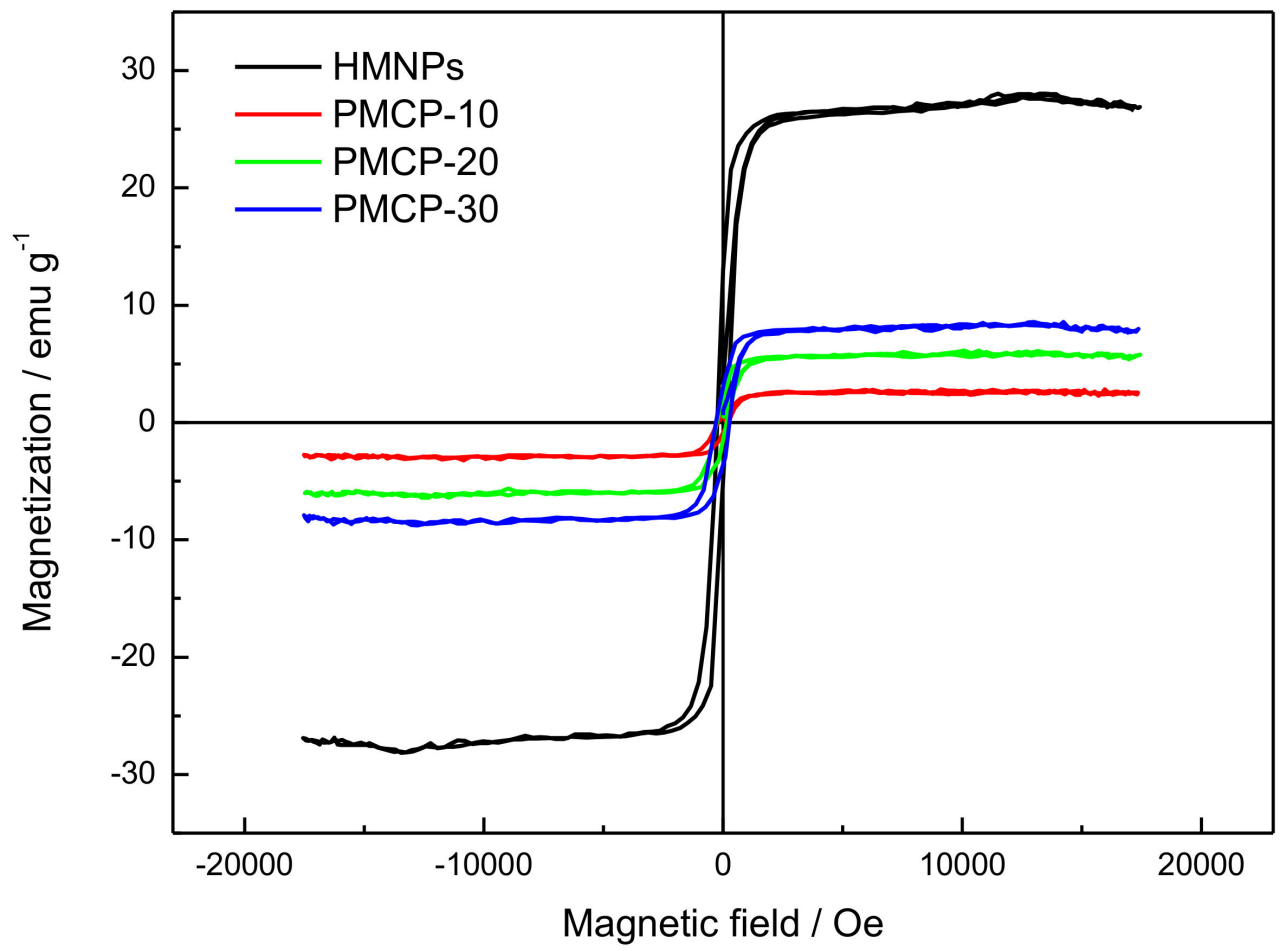
The hysteresis loops of HMNPs and the composite nanoparticles with different HMNP content.

The magnetic hysteresis loops of PMCP-10, PMCP-20, and PMCP-30 were determined in order to investigate their magnetic properties. In Figure 8, the saturated magnetization of pure HMNPs was 26.5 emu/g, while that of the PMCP-10, PMCP-20, and PMCP-30 was measured as 2.6 emu/g, 5.4 emu/g, and 7.7 emu/g, respectively, which were linearly related to their HMNP contents of 10%, 20%, and 30%, suggesting that the HMNPs coated in nanoparticles persisted the original properties of magnetic response. In addition, as analyzed above via the TEM and XRD methods, most HMNPs were superparamagnetic with a particle size below 20 nm, resulting in the exceedingly low coercivity of the samples (indicated by the transverse–axis intercept in Figure 8).

**Figure 9:**
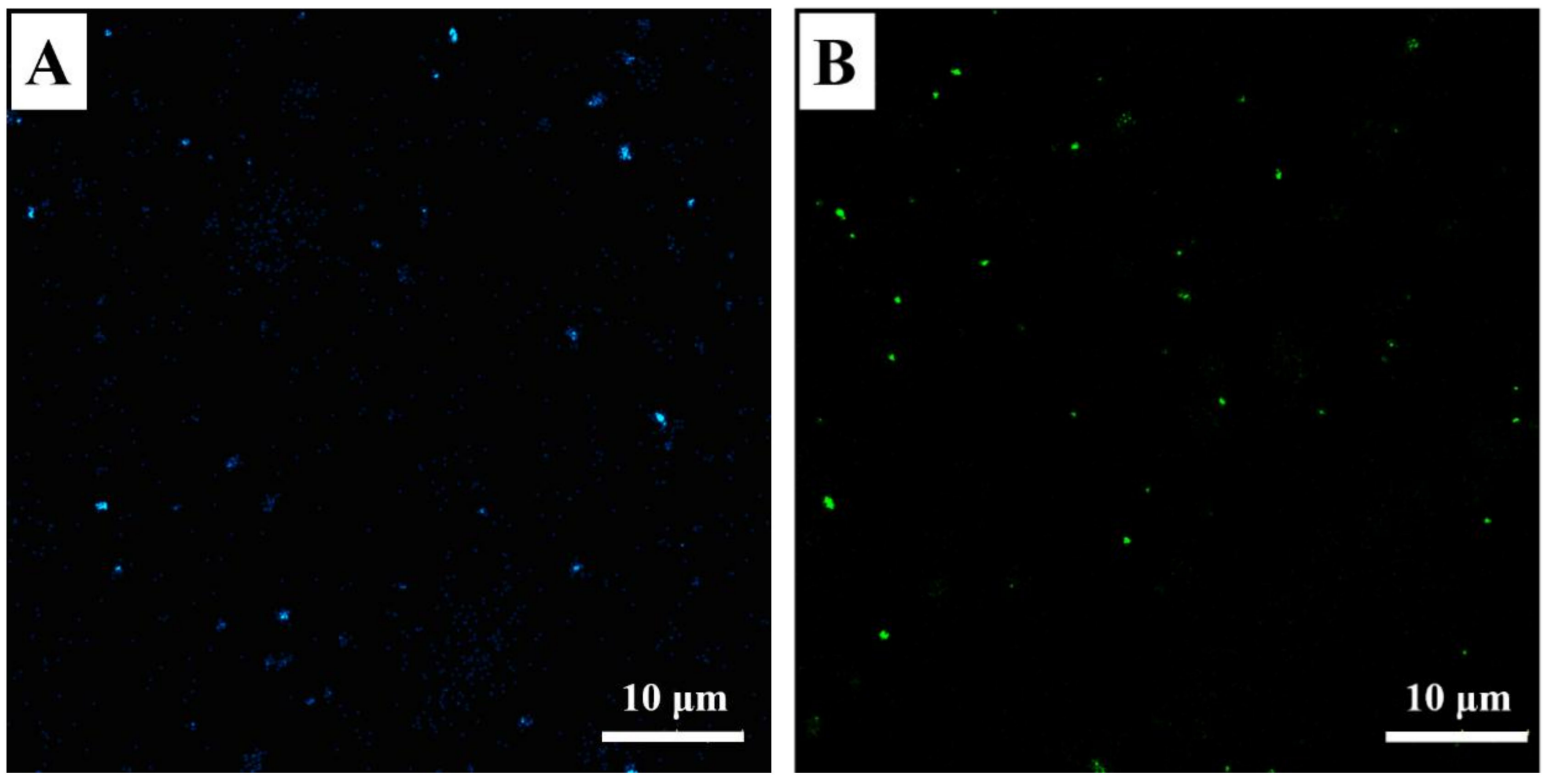
Confocal laser scanning microscopy of (**A**) PMCP-20 and (**B**) PDCP-20.

**Figure 10:**
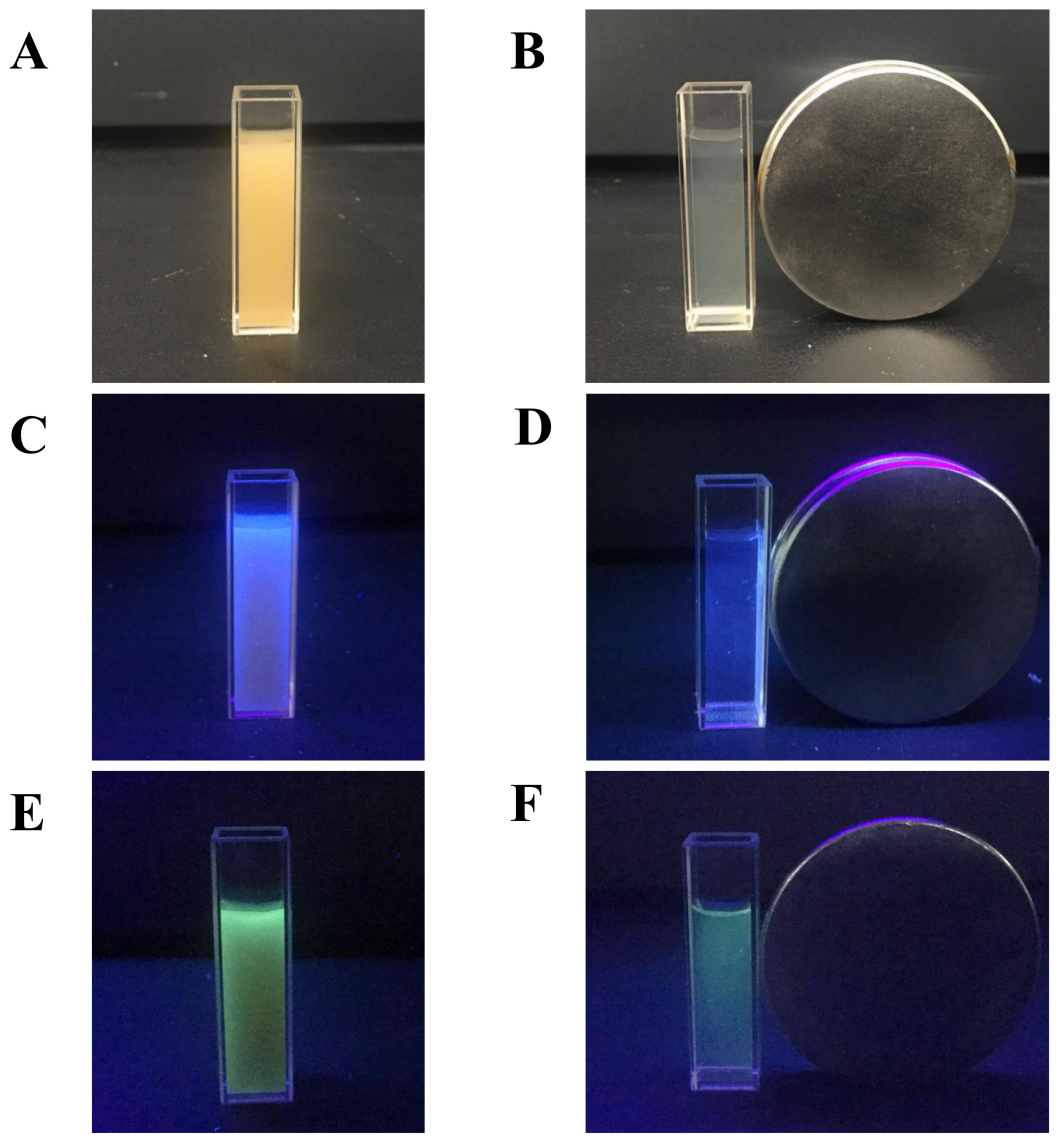
The magnetic response of (**A**,**B**) PMCP-20 latex, (**C**,**D**) PMCP-20 under 365 nm UV light, and (**E**,**F**) PDCP-20 under 365 nm UV light.

Under visible light, both PMCP-20 and PDCP-20 appeared as yellow homogenous latex, whereas their fluorescent colors under UV light corresponded with that of their dye monomers. As shown in Figure 9, the microscopic observation of PMCP-20 and PDCP-20 was conducted by the laser confocal microscope (405 nm exciting light), where the FMJNPs emitting bright fluorescence behaved in an intensive Brownian motion in dilute latex because of their small particle size. Meanwhile, their magnetic response could be directly examined by an external magnetic field (Figure 10). By magnetic attraction, PMCP-20 and PDCP-20 latex gradually became transparent within approximately 16 min (Figure S9), yet the precipitated FMJNPs were able to be quickly re-dispersed by 20 s ultrasonication. In comparison, sodium oleate was also tried as the emulsifier in mini-emulsification to substitute SDS; however, after magnetic attraction, the resulting nanoparticles with zeta potential of −37 mV were unable to be dispersed by sonication. This suggested that the strong ionization of the emulsifier was of importance to generate an adequate negative zeta potential, which triggered the repulsion of the nanoparticles and benefitted reversible dispersion after the removal of the magnetic field.

### 3.5. Cytocompatibility

**Figure 11:**
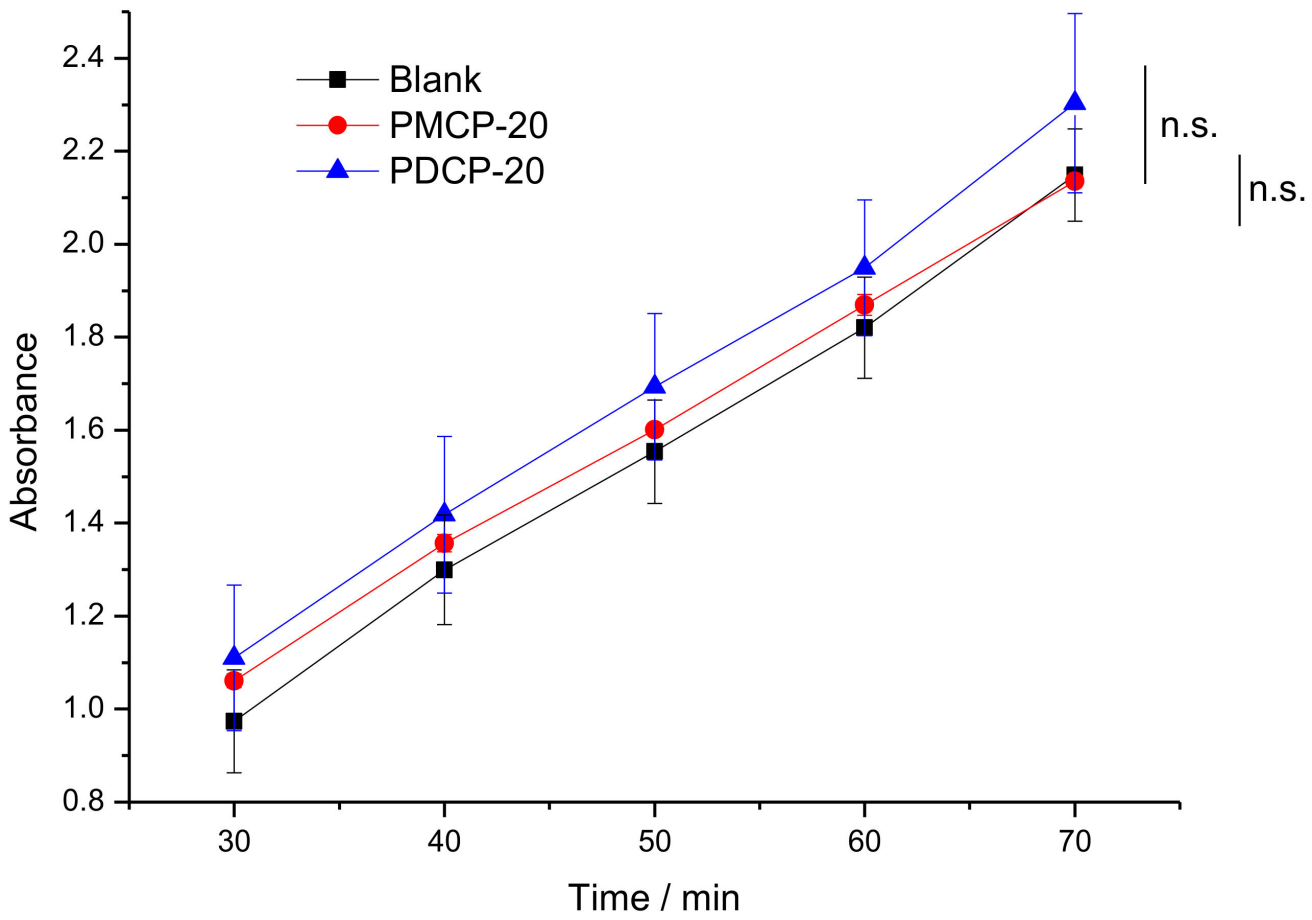
The time–absorbance plot of the blank cell and the cell incubated with PMCP-20 and PDCP-20.

The in vitro cytocompatibility of FMJNPs was determined by the WST-1 method [36]. Herein, the human intestinal epithelial cells were used as model recipients and the cytocompatibility of the nanoparticles was represented by the cell viability and, more exactly, the activity of mitochondrial dehydrogenase in cells. The enzyme could chemically degrade the colorless substrate (WST-1) to a yellow dye. By detecting the absorbance at 450 nm of the yellow product, one can quantitatively measure the activity of mitochondrial dehydrogenase, thus investigating the toxicity or the cytocompatibility of the sample. As illustrated in Figure 11, compared with the blank cell specimen, the sample incubated with PMCP-20 and PDCP-20 (1.5 mg/mL) had relatively similar plots of time–absorbance, indicating that the FMJNPs were nontoxic and had no influence on the cell activity. Despite that the addition of yellow PMCP-20 and PDCP-20 resulted in higher absorbance at 450 nm than the blank sample, the t-test values of PMCP-20 and PDCP-20 against the blank were calculated as 0.432 and 0.143, respectively, being greater than the *p*-value (0.05), so there was statistically considered to be no significant difference (n.s.) between the samples and the blank.

In addition, from the optical microscopy (Figure S10), the cells incubated with PMCP-20 and PDCP-20 maintained normal growth without the collapsed morphology of dead cells, which again supported the outstanding cytocompatibility of the fluorescent magnetic nanoparticles as expected. Accordingly, we envisaged that the FMJNPs prepared by mini-emulsion method might be employed in different areas such as enzyme separation, cell labeling, drug delivery, imaging probe, and so on.

## 4. Conclusions

In this work, two kinds of fluorescent PU prepolymers were firstly synthesized by copolymerization of fluorescent dye monomers MHHNA and DHHNA. It was found that the migration fastness and the fluorescent intensity of fluorophores were improved by the covalent bonding with the polymer chain. Then, the nanoparticles composed of fluorescent PU and hydrophobic Fe_3_O_4_ nanoparticles were efficiently fabricated by mini-emulsification and self-assembly process. Noteworthily, the composition proportion of nonpolar segments of PU played an important role in regulating the nanostructure of products including Janus and core/shell structure, while Janus nanoparticles could be controllably produced as a result of the phase separation between PU and Fe_3_O_4_. By vibrating magnetometer and confocal laser scanning microscope, the prominent magnetic properties and fluorescent properties of the FMJNPs were confirmed. They displayed responses to the magnetic attraction and ultrasonication for reversible aggregation–dispersion behavior. Last but not least, the excellent cytocompatibility of the FMJNPs was proven by the WST-1 method, and the cell incubated with PMCP-20 and PDCP-20 grew normally. Accordingly, these nanoparticles enjoyed potential applications in the biomedical field, and this work might be meaningful for the facile fabrication of various FMJNPs with versatile functions.

## Figures and Tables

**Table 1:** Recipe for the preparation of the polyurethane (PU) prepolymer. MHHNA—4-methoxyl-N-(2-hydroxy-1-hydroxymethylethyl)-1,8-naphthalimide; TMP—trimethylol propane; CO—castor oil; DHHNA—4-diethylamino-N-(2-hydroxy-1-hydroxymethylethyl)-1,8-naphthalimide; IPDI—isophorone diisocyanate; HDO—1,2-hexadecanediol; BDO—1,4-butanediol; THF—tetrahydrofuran.

Prepolymer	IPDI/g	CO/g	TMP/g	HDO/g	BDO/g	Fluorescent dye/g	THF/g
PU-TMP	2.22	0	0.158	0	0.534	0	10
PU-CO	2.22	0.75	0	0	0.592	0	10
PU-MHHNA	2.22	0.4	0	0.2	0.572	0.016 ^a^	10
PU-DHHNA	2.22	0.4	0	0.2	0.572	0.018 ^b^	10

^a^ MHHNA, 5.3 × 10^−2^ mmol. ^b^ DHHNA, 5.3 × 10^−2^ mmol.

**Table 2:** Fabrication formula of composite nanoparticles. HMNPs—hydrophobic magnetic nanoparticles; PTCP—PU-TMP magnetic composite nanoparticle; PCCP—PU-CO magnetic composite nanoparticle; PMCP—PU-MHHNA magnetic composite nanoparticle; PDCP—PU-DHHNA magnetic composite nanoparticle.

Sample	PU Prepolymer/mg		HMNPs/mg
PTCP-20	PU-TMP	80	20
PCCP-20	PU-CO	80	20
PMCP-0	PU-MHHNA	100	0
PMCP-10		90	10
PMCP-20		80	20
PMCP-30		70	30
PDCP-0	PU-DHHNA	100	0
PDCP-10		90	10
PDCP-20		80	20
PDCP-30		70	30

**Table 3:** The colloidal properties of the composite nanoparticles.

Sample	Mean Diameter/nm	Polydis	Zeta Potential/mV
PTCP-20	135.5	0.143	−67.3
PCCP-20	189.0	0.259	−65.1
PMCP-10	125.7	0.052	−69.5
PMCP-20	128.4	0.083	−68.8
PMCP-30	129.0	0.095	−64.1
PDCP-10	130.2	0.064	−59.7
PDCP-20	122.8	0.090	−69.9
PDCP-30	128.8	0.121	−66.8

## Supplementary Materials

The supplementary materials are available online at http://www.mdpi.com/2073-4360/11/2/272/s1.

## Abbreviations

FMNP	fluorescent/magnetic nanoparticle
FMJNP	fluorescent/magnetic Janus nanoparticle
HMNP	hydrophobic magnetic nanoparticle
BHHNA	4-bromo-N-(2-hydroxy-1-hydroxymethylethyl)-1,8-naphthalimide
MHHNA	4-methoxyl-N-(2-hydroxy-1-hydroxymethylethyl)-1,8-naphthalimide
DHHNA	4-diethylamino-N-(2-hydroxy-1-hydroxymethylethyl)-1,8-naphthalimide
PU	polyurethane
TMP	trimethylol propane
CO	castor oil
PU-TMP	polyurethane from trimethylol propane
PU-CO	polyurethane from castor oil
PU-MHHNA	polyurethane from 4-methoxyl-N-(2-hydroxy-1-hydroxymethylethyl)-1,8-naphthalimide
PU-DHHNA	polyurethane from 4-diethylamino-N-(2-hydroxy-1-hydroxymethylethyl)-1,8-naphthalimide
PTCP	PU-TMP magnetic composite nanoparticle
PCCP	PU-CO magnetic composite nanoparticle
PMCP	PU-MHHNA magnetic composite nanoparticle
PDCP	PU-DHHNA magnetic composite nanoparticle
HDO	1,2-hexadecanediol
BDO	1,4-butanediol
NA	1,8-naphthalimide
SDS	sodium dodecyl sulfate
IPDI	isophorone diisocyanate
THF	tetrahydrofuran
DMSO	dimethyl sulfoxide
XRD	X-ray diffraction
TEM	transmission electron microscopy
SEM	scanning electron microscopy
DLS	dynamic light scattering
GPC	gel permeation chromatography
