# Supplementary material for: Synthesis and Morphological Control of Biocompatible Fluorescent/Magnetic Janus Nanoparticles Based on the Self-Assembly of Fluorescent Polyurethane and Fe3O4 Nanoparticles
Source: Polymers (Basel). 2019 Feb 5;11(2):272. doi: 10.3390/polym11020272 (PMC6419061; doi:10.3390/polym11020272)
Supplement: Supplementary file 1 [file polymers-11-00272-s001.pdf]

Supplementary information

Synthesis and Morphological Control of Biocompatible Fluorescent/Magnetic Janus Nanoparticles Based on the Self-Assembly of Fluorescent Polyurethane and Fe_3O_4 Nanoparticles

Botian Li [1,4], Wei Shao [1], Yanzan Wang [1], Da Xiao [1], Yi Xiong [3], Haimu Ye [1], Qiong Zhou [1,*], Qingjun Jin [2,4,*]

[1] Department of Materials Science and Engineering, China University of Petroleum, Beijing, 102249, P. R. China; botian.li@cup.edu.cn (B.L.); shao222333@sina.com (W.S.); wangyanzan_cup@163.com (Y.W.); 18811372167@163.com (D.X.); yehaimu@cup.edu.cn (H.Y.)

[2] Research Institute of Chemical Defense, Academy of Military Sciences, Beijing, 102205, P. R. China.

[3] College of Safety and Ocean Engineering, China University of Petroleum, Beijing, 102249, P. R. China; xiongyi@cup.edu.cn (Y.X.)

[4] Key Laboratory of Advanced Materials of Ministry of Education of China, Department of Chemical Engineering, Tsinghua University, 100084, P. R. China.

* Correspondence: zhouqiong_cn@163.com (Q.Z.); jinqingjun502@163.com (Q.J.); Tel.: +86-10-8973-3200

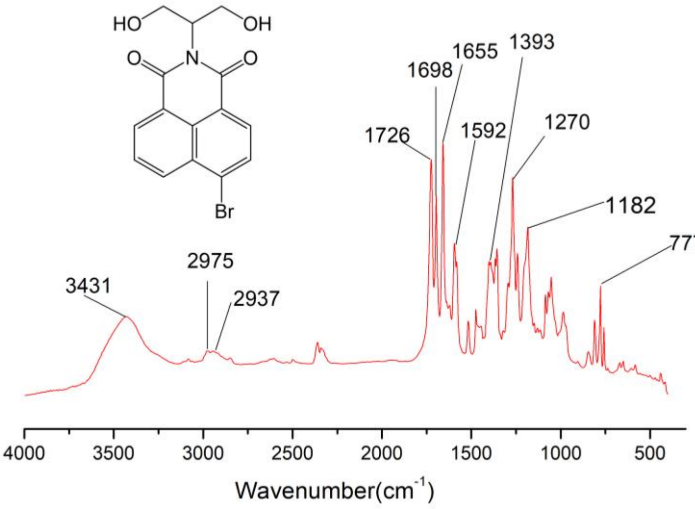

Figure S1. FT-IR spectroscopy of BHHNA.

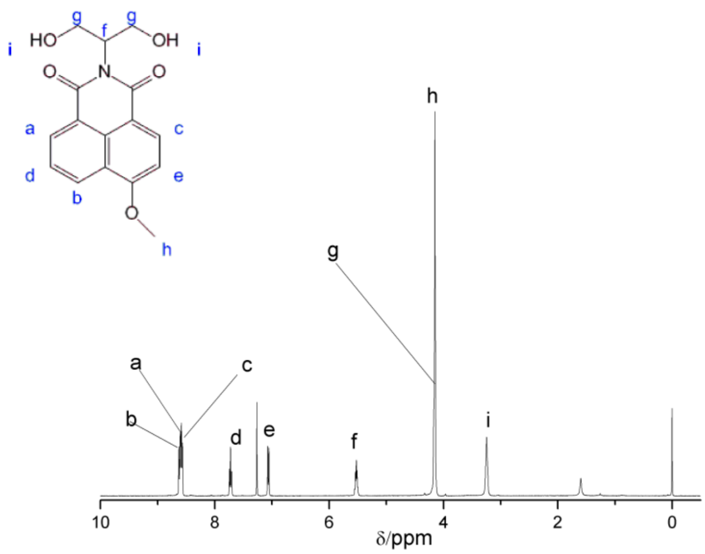

Figure S2. ¹H NMR spectrum of MHHNA.

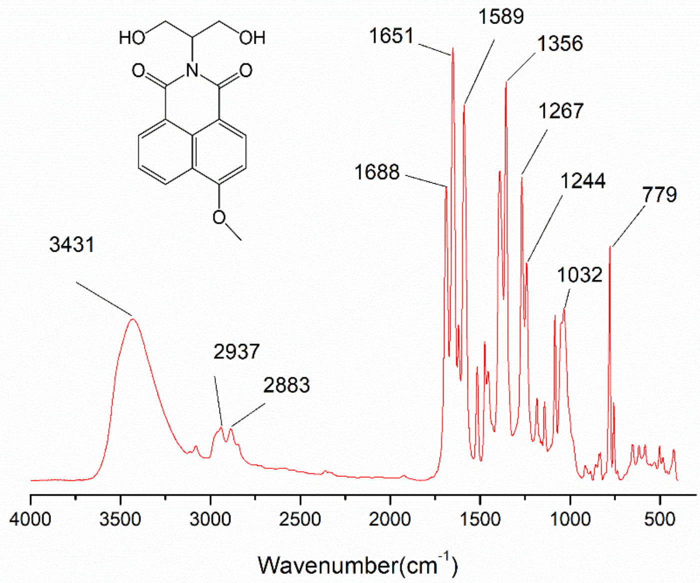

Figure S3. FT-IR spectroscopy of MHHNA.

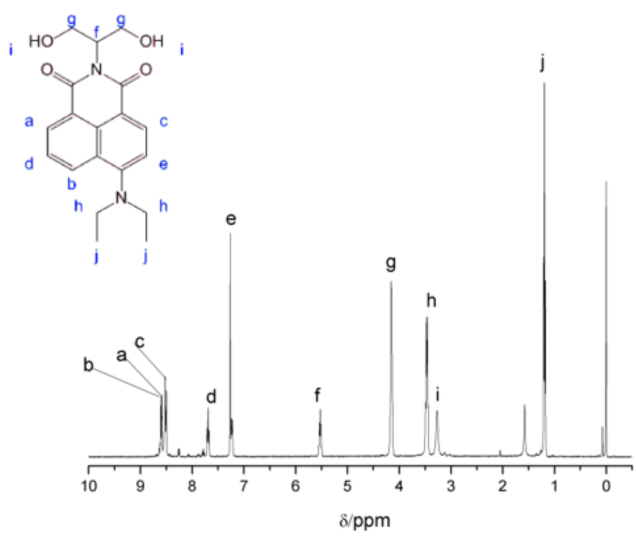

Figure S4. ^{1}H NMR spectrum of DHHNA.

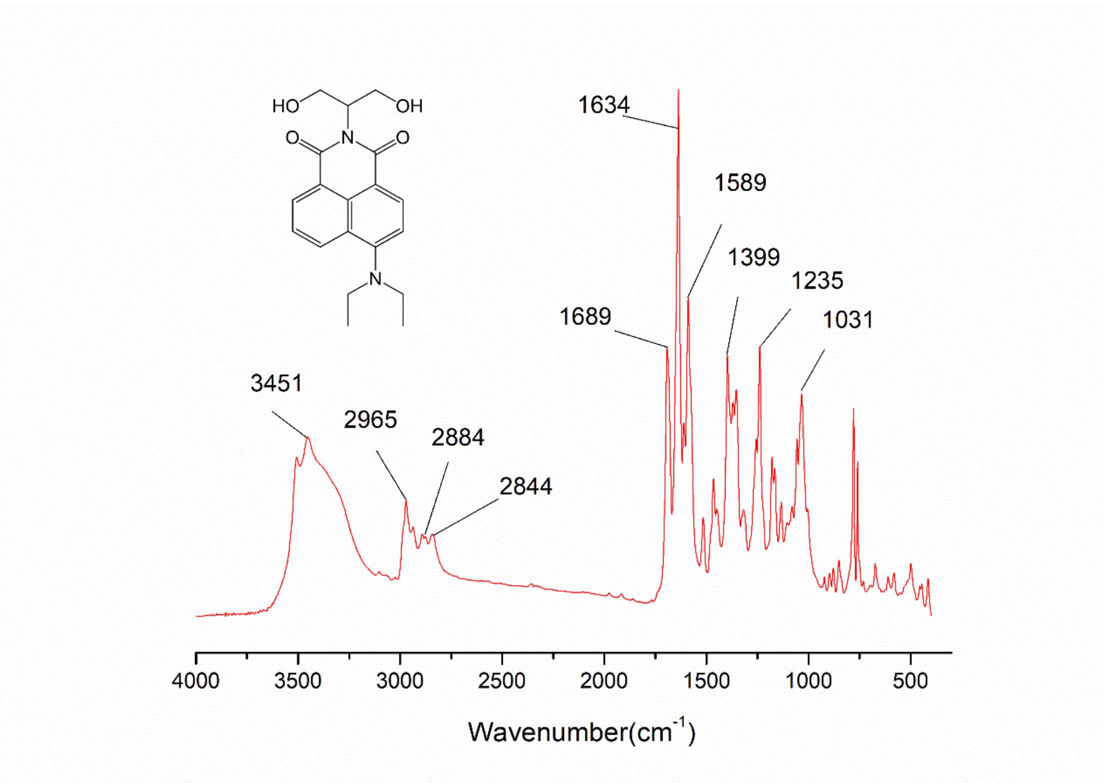

Figure S5. FT-IR spectroscopy of DHHNA.

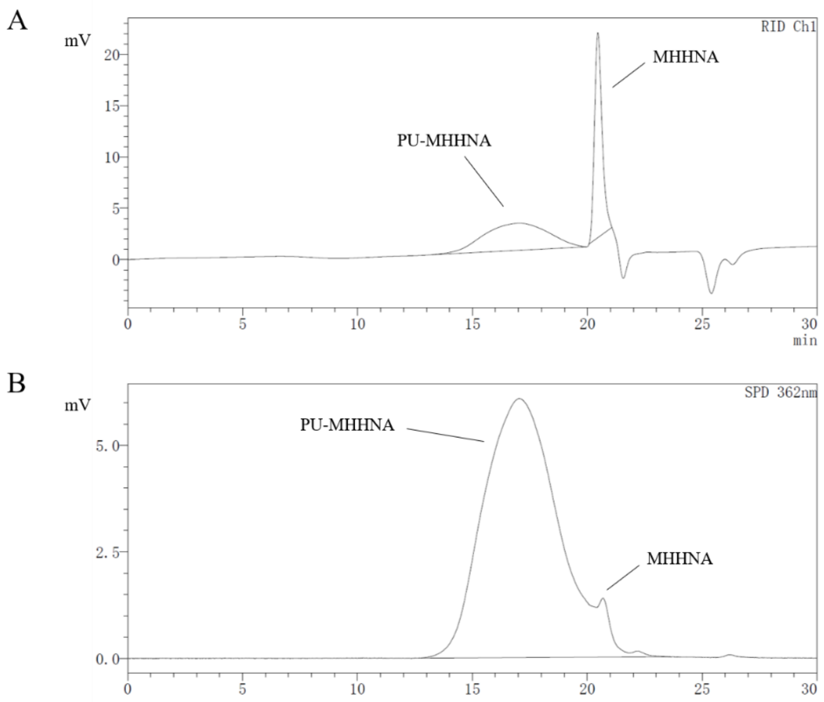

Figure S6. GPC analysis of PU-MHHNA (**A**) with a refractive index detector and (**B**) with an UV-Vis detector.

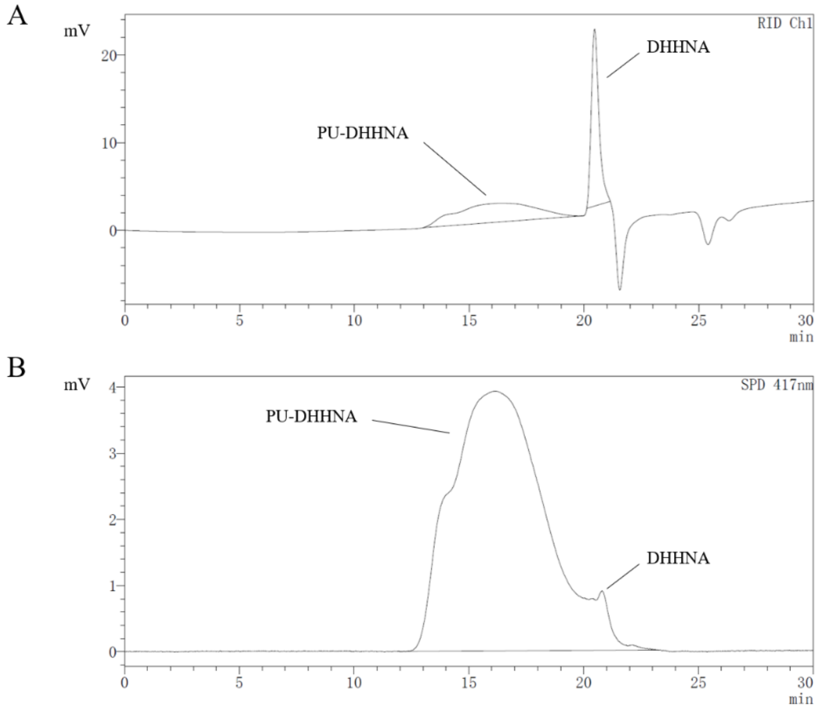

Figure S7. GPC analysis of PU-DHHNA (**A**) with a refractive index detector and (**B**) with an UV-Vis detector.

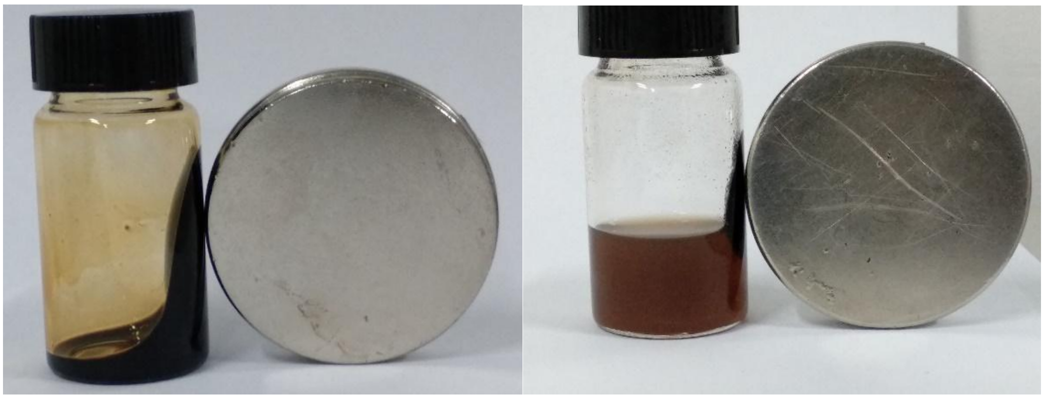

Figure S8. Photographs of the Fe_3O_4 nanoparticles dissolved in good solvent (toluene) (left) and precipitated in poor solvent (ethanol) (right) with an external magnetic field.

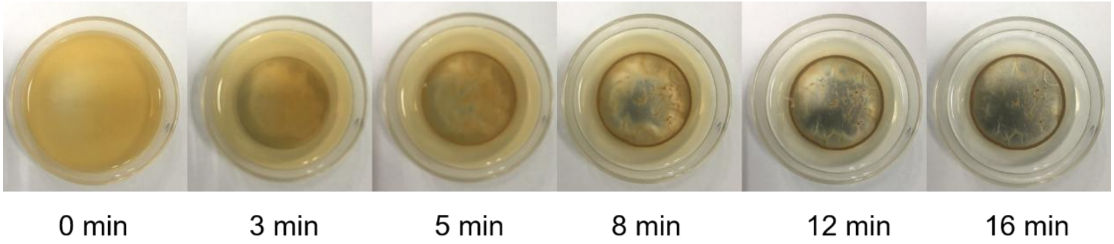

Figure S9. Photographs of the PDCP-2 latex with a magnet underneath at different times.

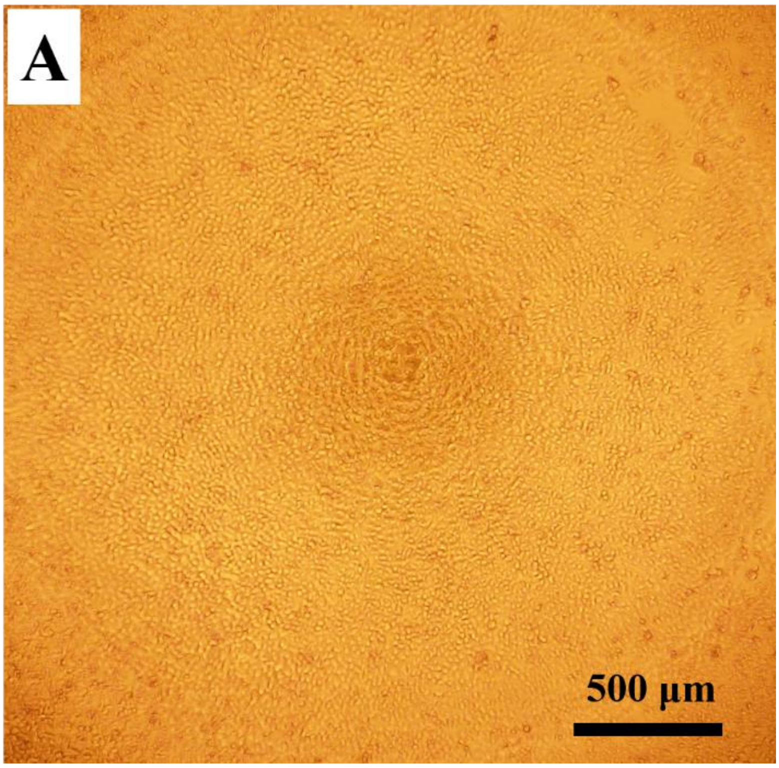

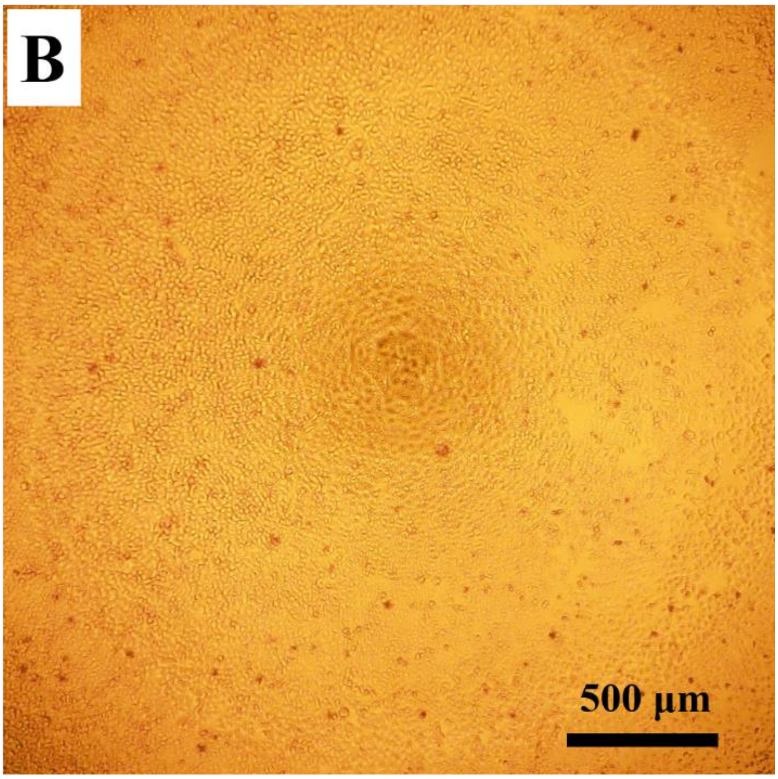

Figure S10. Optical microscopy of the dyed cells after 80 min incubation with (**A**) PMCP-2 and (**B**) PDCP-2.
